# Brain Metastases in Gastrointestinal Cancers: Is there a Role for Surgery?

**DOI:** 10.3390/ijms150916816

**Published:** 2014-09-22

**Authors:** Johannes Lemke, Jan Scheele, Thomas Kapapa, Silvia von Karstedt, Christian Rainer Wirtz, Doris Henne-Bruns, Marko Kornmann

**Affiliations:** 1 Clinic of General and Visceral Surgery, University Hospital Ulm, Albert-Einstein-Allee 23, Ulm 89071, Germany; E-Mails: johanneslemke@gmx.de (J.L.); jan.scheele@uniklinik-ulm.de (J.S.); doris.henne-bruns@uniklinik-ulm.de (D.H.-B.); 2 Clinic of Neurosurgery, University Hospital Ulm, Albert-Einstein-Allee 23, Ulm 89071, Germany; E-Mails: thomas.kapapa@uniklinik-ulm.de (T.K.); rainer.wirtz@uniklinik-ulm.de (C.R.W.); 3 UCL Cancer Institute, University College London, 72 Huntley Street, London WC1E 6DD, UK; E-Mail: s.karstedt@ucl.ac.uk

**Keywords:** brain metastases, gastrointestinal cancer, esophageal cancer, gastric cancer, pancreatic cancer, colorectal cancer

## Abstract

About 10% of all cancer patients will develop brain metastases during advanced disease progression. Interestingly, the vast majority of brain metastases occur in only three types of cancer: Melanoma, lung and breast cancer. In this review, we focus on summarizing the prognosis and impact of surgical resection of brain metastases originating from gastrointestinal cancers such as esophageal, gastric, pancreatic and colorectal cancer. The incidence of brain metastases is <1% in pancreatic and gastric cancer and <4% in esophageal and colorectal cancer. Overall, prognosis of these patients is very poor with a median survival in the range of only months. Interestingly, a substantial number of patients who had received surgical resection of brain metastases showed prolonged survival. However, it should be taken into account that all these studies were not randomized and it is likely that patients selected for surgical treatment presented with other important prognostic factors such as solitary brain metastases and exclusion of extra-cranial disease. Nevertheless, other reports have demonstrated long-term survival of patients upon resection of brain metastases originating from gastrointestinal cancers. Thus, it appears to be justified to consider aggressive surgical approaches for these patients.

## 1. Introduction

Despite considerable progress in diagnostics and therapy, successfully treating cancer still remains one of the major challenges in medicine today since (i) incidence is increasing due to aging and growth of the world’s population and (ii) cancer still remains a leading cause of death worldwide [1]. At diagnosis, patients are initially staged for local progression and systemic spread (metastasis) of their disease which, together, determine whether a curative therapeutic approach is considered or not. In the majority of cases, complete surgical resection of the primary tumor is the key to attempt curative treatment. Recent studies revealed that prognosis of curatively treated patients can sometimes be further improved by multimodal treatment, including chemo- and radiotherapy. Thus, in patients with locally controlled disease, long-term survival and/or cure is frequently achieved today for many cancer entities. Being the cause for most cancer-related deaths, treatment of metastatic disease remains a major challenge in cancer therapy today [2].

Brain metastases are frequently discovered in cancer patients and, in fact, intracranial metastatic lesions are far more common than primary brain tumors [3]. About 10% of all cancer patients will develop brain metastases during cancer progression [4]. However, the likeliness of their development largely depends on the primary tumor entity since the vast majority of brain metastases arise from only three human cancers: melanoma, lung- and breast cancer [5]. Within these three entities, brain lesions are most often diagnosed in patients with lung cancer, whilst melanoma patients bear the highest risk to developing intracranial lesions [5]. However, despite the increased incidence in melanoma, breast and lung cancer, occurrence of metastatic spread to the brain has been described for all types of cancer. Although cancers originating from the gastrointestinal tract frequently metastasize to distant sites, the brain appears to be a very rare location for metastases originating from these entities. Our comprehensive analysis of clinical studies which investigated incidence and outcome of brain metastases originating from different gastrointestinal cancers confirmed a very low incidence. Moreover, it revealed differences within the different gastrointestinal cancer entities discussed here. Whilst the incidence of brain metastasis originating from gastric and pancreatic cancer is below 1%, its incidence is reported to be as high as 4% in esophageal and colorectal cancer (Table 1). The reasons for these differences remain unclear. However, it is likely that different genetic make-ups of the tumor entities as well as diverging mechanisms and routes of hematological dissemination may contribute to this fact.

Whilst metastases located in several organs can often be asymptomatic for a long time, brain metastases commonly become symptomatic early on, causing nausea, headache and/or neurological deficits, demanding prompt and aggressive treatment [6]. Brain metastases-targeting therapy may be applied to relieve these symptoms but also to decrease mortality and/or to achieve a cure in some cases. These therapeutic options include neurosurgery, whole brain radiotherapy (WBRT), stereotactic radio-surgery and chemotherapy, both singular or combined [7].

**Table 1:** Incidence of brain metastases in gastrointestinal cancer.

Cancer	New Cases per Year [8]	Incidence BM
Esophageal	482,000	1.4%–3.9%
Gastric	989,000	0.2%–0.7%
Pancreatic	277,000	0.3%
Colorectal	1,233,000	0.6%–4.0%

BM = brain metastases.

Overall, prognosis of patients diagnosed with brain metastases is poor with a median survival in the range of a few months [6,9]. A large study analyzing the outcome of 1292 patients diagnosed with brain metastases found a median survival of only 3.4 months [9]. However, since the 1990s there is an increasing body of evidence that surgical resection of brain metastases can significantly decrease morbidity and prolong survival as compared to non-surgical approaches [7,10,11]. Importantly, age and patient performance status as well as the number of brain metastases, occurrence of extra-cranial metastases and the primary tumor site were found to be important prognostic factors in patients with brain metastases [12,13,14]. However, due to relatively few numbers of clinical studies (and patients), validations of these prognostic criteria for patients diagnosed with brain metastases from gastrointestinal tumors is largely missing. Consequently, standard recommendations how to treat these patients have not been established yet. In this review, clinical studies of the last 15 years about brain metastases in esophageal, gastric, pancreatic and colorectal cancer are summarized with a focus on prognosis and the role of surgery in the therapy of brain metastasis in these cancer entities.

## 2. Brain Metastases of Gastrointestinal Cancers

### 2.1. Esophageal Cancer

Esophageal cancer is a relatively rare cancer entity to date; however its incidence is rapidly increasing [15]. Its highest prevalence is found in Asian countries, presumably due to environmental factors such as food and lifestyle choices [16]. Esophageal cancer is histo-pathologically classified into two major forms, squamous cell carcinomas and adenocarcinomas. Squamous cell carcinomas are thought to originate from inflammation-driven dysplasia and are usually found in the upper two thirds of the esophagus. In contrast, adenocarcinomas are usually localized in the distal esophagus and arise from metaplastic epithelium, also known as Barrett’s esophagus, which is caused by chronic inflammation driven by gastroesophageal reflux disease (GERD) [17]. The prognosis for patients with esophageal cancer remains poor with a 5-year-overall survival rate of 10%–15% for both histological types [18]. Surgical resection provides the only chances for cure, which is, however, only performed in one fourth of all patients. Nowadays, patients often receive neo(adjuvant) multimodal therapy and show improved prognosis with a 5-year-overall survival rate of about 30% [19]. Most patients, however, are diagnosed only at a late and advanced tumor stage, which is defined by tumor infiltration of adjacent tissues and/or evidence for distant metastases. In these cases, therapy remains mostly palliative including radio-, brachy-, and chemotherapy. If distant metastases are diagnosed, they are mostly detected in the liver, lung, or adrenal glands [20]. Brain metastases occur with a frequency of 1.4%–3.9% in esophageal cancer and are therefore a rare phenomenon (Table 2). The largest studies by Weinberg and colleagues and Song and colleagues included 1588 patients and 1612 patients with esophageal cancer, respectively. They found that only 1.7% and 1.6% developed brain metastases during the course of their disease. Interestingly, Song *et al.* suggested that the incidence of brain metastases might be higher in squamous cell carcinomas as compared to adenocarcinomas [21]. Most patients with brain metastases from esophageal cancer were male, in line with the increased incidence of this cancer in male patients [16]. Moreover, the incidence of brain metastases in esophageal cancer was associated with an advanced tumor stage and with the prevalence of additional extra-cranial metastases. In about half of all cases, multiple brain metastases were found. The median survival of all patients diagnosed with brain metastases from esophageal cancer was in most cases less than 5 months (Table 2). Interestingly, therapeutic approaches involving surgical resections were associated with improved survival (Table 2). Along these lines, Ogawa and colleagues identified five long term survivors in their cohort with survival times of more than one year after receiving surgical resection plus radiotherapy. Of note, all of them were in a good state of health, none of them presented with extra-cranial disease and the majority was diagnosed with a solitary intracranial lesion [22]. Another interesting study suggested that esophageal cancer patients who receive adjuvant therapy show an increased incidence of brain metastases, which, however, cannot be solely explained by prolonged survival of these patients [23]. Whether screening of those patients for the development of brain metastases is reasonable remains controversial since another study suggested no benefit for screening or surveillance in this context [24]. In summary, brain metastases originating from esophageal cancer are very rare, usually develop and/or are detected at advanced tumor stage, and are associated with poor survival. However, there appears to be some evidence that aggressive treatment including surgical resection may prolong survival in selected patients, in particular those who are diagnosed with solitary brain metastases and do not show additional, extra-cranial metastatic disease.

**Table 2:** Clinical studies analyzing characteristics and survival of patients diagnosed with brain metastases originating from esophageal cancer.

Inc	*n*	Age (Years)	Male	ECM	BM > 1	Resection	OS (Months)	OS Resection (Months)	Reference
1.4%	36	62	92%	53%	53%	33%	3.9	9.6	[22]
1.7%	27	62	100%	70%	52%	37%	3.8	3.8–9.6	[25]
3.4%	29	n/a	n/a	44%	52%	21%	3.5	improved	[23]
2.1%	17	58	n/a	76%	35%	59%	n/a	improved	[26]
2.7%	12	64	92%	75%	67%	17%	2.1	1.2–7.0	[27]
3.9%	20	n/a	n/a	45%	40%	60%	10.5	n/a	[24]
1.6%	26	62	96%	69%	54%	19%	4.2	7.0	[21]

Inc = Incidence of brain metastases in the respective study; *n* = number of patients enrolled in the study; Male = Percentage of male patients of the patient cohort; ECM = percentage of patients diagnosed with extra-cranial metastases; BM > 1 = percentage of patients diagnosed with more than one brain metastases; Resection = percentage of patients that received surgical resection of brain metastases; OS = median survival of all patient diagnosed with brain metastases within the respective study; OS Resection = median survival of the patients who received surgical resection of brain metastases; n/a = data not available.

### 2.2. Gastric Cancer

Gastric cancer is one of the most frequent cancers with almost 1,000,000 new cases per year worldwide [8]. Adenocarcinomas are the most common histo-pathological type and its origin is thought to be multi-factorial, including infection (e.g., helicobacter pylori or Ebstein-Barr virus), alimentation, smoking and use of drugs modulating gastric ambience, such as NSAIDs [28]. Surgical resection provides a chance for cure [29] and neoadjuvant as well as adjuvant chemo (radio) therapy have been shown to further improve patient outcome [30]. However, the majority of newly diagnosed gastric carcinomas are considered unresectable due to locally advanced tumor growth or distant metastases, which in many cases involve the liver [31]. The prognosis of these patients is very poor with a 5-year-overall survival rate below 5% [32]. Only a very few reports have focused on metastases to the central nervous system in gastric cancer (Table 3). These reports revealed an incidence below 1%, which is, interestingly, markedly lower than the incidence of brain metastases in esophageal cancer, despite its anatomical proximity. Respective studies found that if brain metastases developed, most patients were found to already have systemic spread to other organs. Moreover, in about half of these patients, multiple brain metastases were found. The reported overall survival rate ranged from 1.4 to 27.7 months. Interestingly, all studies found that aggressive treatment comprising surgical resection of brain metastases could improve survival of these patients.

**Table 3:** Clinical studies analyzing characteristics and survival of patients diagnosed with brain metastases originating from gastric cancer.

Inc	*n*	Age (Years)	Male	ECM	BM > 1	Resection	OS (Months)	OS Resection (Months)	Reference
0.7%	24	53	75%	88%	55%	21%	2.4	12.5	[33]
0.2%	11	44	54%	n/a	n/a	0%	1.4–2.2	n/a	[34]
0.5%	11	55	82%	20%	55%	27%	2.7	6	[35]
n/a	11	61	64%	82%	45%	18%	27.7	45.5	[36]
n/a	56	56	79%	91%	55%	n/a	2.1–9.3	n/a	[37]

Inc = Incidence of brain metastases in the respective study; *n* = number of patients enrolled in the study; Male = Percentage of male patients of the patient cohort; ECM = percentage of patients diagnosed with extra-cranial metastases; BM > 1 = percentage of patients diagnosed with more than one brain metastases; Resection = percentage of patients that received surgical resection of brain metastases; OS = median survival of all patient diagnosed with brain metastases within the respective study; OS Resection = median survival of the patients who received surgical resection of brain metastases; n/a = data not available.

### 2.3. Pancreatic Cancer

Pancreatic cancer is one of the deadliest cancers demonstrated by the fact that its incidence almost equals its mortality [8]. The 5-year-overall survival rate is only about 5% [38]. In most cases, pancreatic cancers are adenocarcinomas arising from the pancreatic ductal epithelium (pancreatic ductal adenocarcinomas). This malignancy presents with very aggressive disease features including invasiveness and early metastatic spread [39]. In most cases, pancreatic cancer is only diagnosed when it has already reached a state where its complete surgical removal cannot be achieved anymore (unresectable disease) and/or distant metastases are already present (systemic disease) [40]. Although palliative treatment protocols have been established over the last decades, median survival of patients with metastatic pancreatic cancer is only about 6 months [41]. Pancreatic cancer most frequently metastasizes to the liver since it is drained by the portal vein [42]. Thus, metastatic dissemination of pancreatic cancer to the central nervous system is extremely rare. To date, only one study systematically analyzed the incidence and prognosis of brain metastases in pancreatic cancer (Table 4). Interestingly, of 1229 pancreatic cancer patients followed-up, only four developed brain metastases (0.3%) [43]. All of these patients also presented with metastases at additional sites. None of them was eligible (and/or considered) for surgical treatment and their clinical course was fatal with a median survival of 2.9 months. In addition to this study, only a small number of case reports described this unusual site of pancreatic cancer metastases [44]. Due to the very small number of reported cases, the value of any treatment strategy for brain metastases arising from pancreatic cancer remains unsolved. In summary, the prognosis of patients presenting with brain metastases appear to be even worse as compared to the outcome of patients with metastatic pancreatic cancer in general. However, we previously reported on two cases which demonstrated that long-term survival can be achieved by radical surgical treatment of these metastases [45]. Of note, both patients had a good performance status, presented with symptomatic and solitary brain metastases and without evidence for further extra-cranial disease.

**Table 4:** Clinical studies analyzing characteristics and survival of patients diagnosed with brain metastases originating from pancreatic cancer.

Inc	*n*	Age (Years)	Male	ECM	BM > 1	Resection	OS (Months)	OS Resection (Months)	Reference
0.3%	4	51.5	100%	100%	50%	0%	2.9	n/a	[43]

Inc = Incidence of brain metastases in the respective study; *n* = number of patients enrolled in the study; Male = Percentage of male patients of the patient cohort; ECM = percentage of patients diagnosed with extra-cranial metastases; BM > 1 = percentage of patients diagnosed with more than one brain metastases; Resection = percentage of patients that received surgical resection of brain metastases; OS = median survival of all patient diagnosed with brain metastases within the respective study; OS Resection = median survival of the patients who received surgical resection of brain metastases; n/a = data not available.

**Table 5:** Clinical studies analyzing characteristics and survival of patients diagnosed with brain metastases originating from colorectal cancer.

Inc	*n*	Age (Years)	Male	ECM	BM > 1	Resection	OS (Months)	OS Resection (Months)	Reference
n/a	73	62	41%	n/a	11%	100%	8.3	8.3	[46]
1.6%	17	59	76%	88%	n/a	6%	4.5	n/a	[47]
n/a	32	62	66%	n/a	28%	88%	7.5	10.6	[48]
n/a	49	66	67%	82%	53%	31%	5.1	5.2	[49]
n/a	30	66	60%	87%	27%	100% *	n/a	5.5	[50]
2.3	39	59	54%	97%	n/a	41%	n/a	improved	[51]
n/a	35	59–65	n/a	56%–78%	44%–56%	12%–22%	3.0–5.0	9.0	[52]
0.6%	27	66	52%	93%	56%	26%	2.4	n/a	[53]
1.4%	126	62	62%	91%	60%	16%	5.4	11.5	[54]
n/a	118	54	53%	90%	50%	20%	4.1	7.2	[55]
0.7	60	63	60%	88%	65%	13%	8.0	n/a	[56]
n/a	78	n/a	39%	64%	n/a	25%	8.0	14.0	[57]
4.0%	52	61	56%	90%	27%	12%	3.2	13.2	[58]
1.3%	29	58	79%	79%	69%	59%	6.4	8.8	[59]
n/a	39	59	59%	97%	38%	15%	5.0	15.2	[60]
n/a	48	63	52%	90%	70%	38%	4.0	3.0–13.0	[61] *
1.1%	47	n/a	n/a	n/a	n/a	23%	6.6	16.2	[62]
n/a	28	62	46%	53%	18%	100% *	n/a	12.0	[63]
2.7%	113	n/a	65%	78%	44%	56%	5.4	15.2	[64]
n/a	41	58	61%	95%	41%	29%	5.0	21.4	[65]

Inc = Incidence of brain metastases in the respective study; *n* = number of patients enrolled in the study; Male = Percentage of male patients of the patient cohort; ECM = percentage of patients diagnosed with extra-cranial metastases; BM > 1 = percentage of patients diagnosed with more than one brain metastases; Resection = percentage of patients that received surgical resection of brain metastases; OS = median survival of all patient diagnosed with brain metastases within the respective study; OS Resection = median survival of the patients who received surgical resection of brain metastases; n/a = data not available; * manuscript was not identified by literature search using the MeSH terms indicated but subsequently during manuscript preparation.

### 2.4. Colorectal Cancer

Colorectal cancer is the most frequently encountered cancer of the gastrointestinal tract. It is the third most common cancer in men (following prostate and lung cancer) and second most common in women (following breast cancer). Major risk factors for sporadic colorectal cancers are age and a family history of colorectal cancer. In addition, environmental factors are currently thought to contribute to the development of colorectal cancer [66]. Survival of patients with colorectal cancer is notably better compared to patients diagnosed with other gastrointestinal cancers (5-year-overall survival of about 65%). However, survival mainly depends on the diagnosed tumor stage [67]. Distant metastases are mainly detected in the liver and the lung. At that stage (stage IV colorectal cancer), the 5-year-overall survival rate drops to 8% [68]. In contrast to other gastrointestinal cancer entities discussed above, a substantial number of studies have addressed the incidence and prognosis of brain metastases in colorectal cancer (Table 5). The incidence of brain metastases in colorectal cancer patients was found to be 1.3% up to 4%. In the vast majority, concomitant metastatic spread to other organs was found in these patients. Interestingly, Bryne and co-workers investigated the incidence of brain metastases after resection of colorectal liver metastases and found that only 4% of these patients will subsequently develop brain metastases [58]. The occurrence of multiple brain metastatic lesions as compared to solitary brain metastases seems to largely depend on the study, ranging from 18% up to 70% (Table 5). The overall survival of colorectal cancer patients diagnosed with brain metastases is reported to be 3.2 up to 8.3 months which is poor given a median survival rate of 15–21 months of patients with colorectal cancer metastatic to the liver and/or lung receiving palliative chemotherapy [69]. Interestingly, a number of studies found that neurosurgical removal of brain metastases from colorectal cancers offered the chance for prolonged survival (Table 5). This beneficial effect was maintained despite presence of additional extra-cranial metastatic lesions [60]. Intriguingly, one study found that additional postsurgical radiotherapy can further and significantly increase the survival of patients who received surgical treatment of brain metastases [50]. In all of these studies, it remains widely unclear on which basis patients were selected for surgical resection, and furthermore, which subgroup of this patient cohort actually took advantage of such an aggressive therapeutic approach. However, it is noteworthy that in studies that only enrolled surgically treated patients, patients usually only presented with a solitary intracranial metastatic lesion [46,50,63]. Furthermore, in the study of Wronski and Arbit, which reported on 73 patients that all underwent surgical resection of brain metastases originating from colorectal cancer, only patients were enrolled that were diagnosed with up to two intracranial lesions and with “limited systemic disease” [46]. These figures indicate that patients that are selected for surgical resection are often already associated with positive prognostic factors which may contribute to their improved outcome after surgical resection of brain metastases. In summary, for colorectal cancer a substantial body of data about incidence and prognosis of patients with brain metastases is available. These studies demonstrate that the overall incidence is low, however, higher than in pancreatic or gastric cancer. The prognosis of patients with brain metastases is poor, and is considerably worse than the prognosis of metastatic colorectal cancer in general. However, there is some evidence that aggressive treatment comprising surgical approaches may be beneficial for survival in a subset of patients.

**Figure 1:**
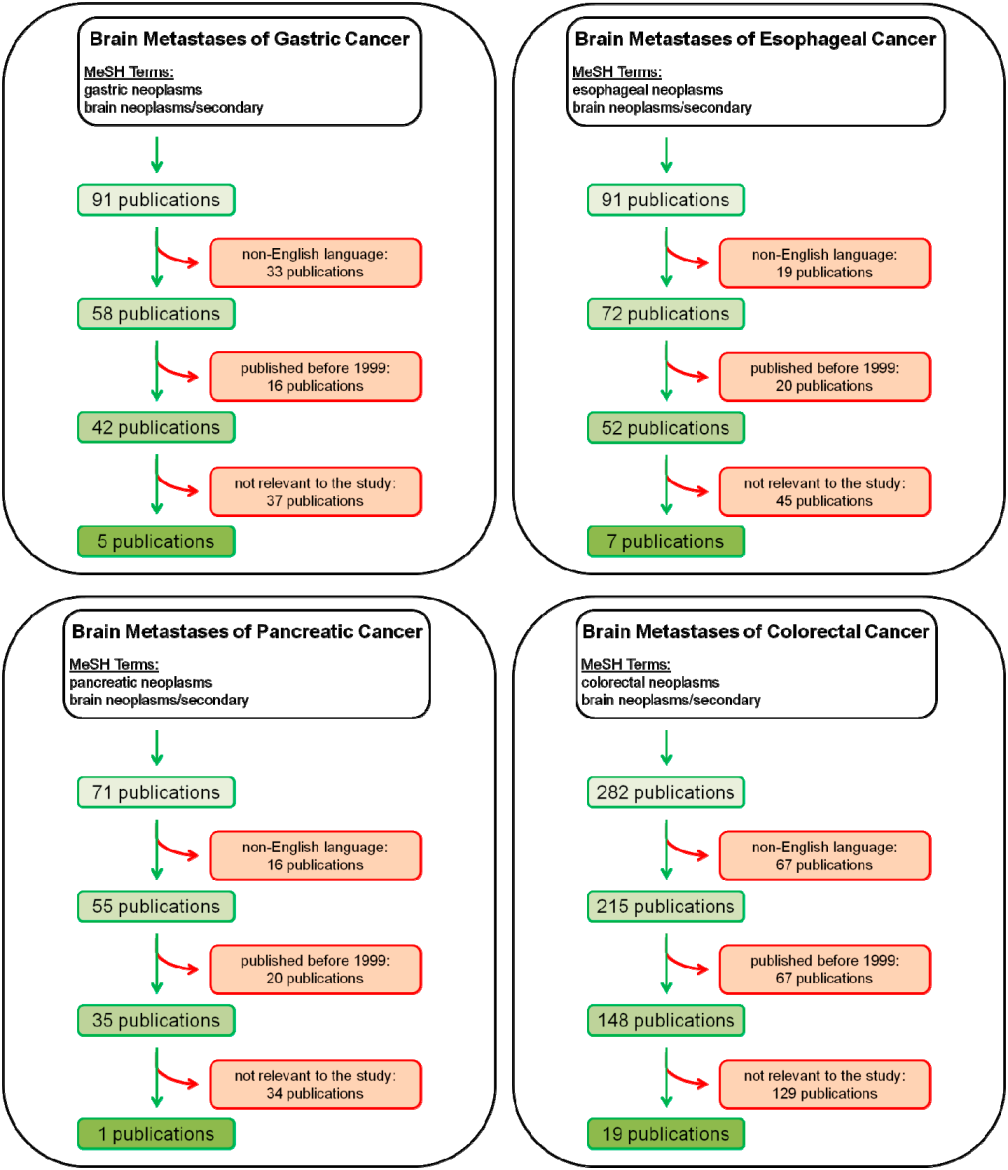
Flow charts of the literature search are provided. The search terms for the Medline query as well as the numbers of publications included and excluded for esophageal, gastric, pancreatic and colorectal cancer are shown.

## 3. Materials and Methods

A Medline query was performed for the MeSH terms “brain neoplasms/secondary” and “esophageal neoplasms”, “gastric neoplasms”, “pancreatic neoplasms” and “colorectal neoplasms”, respectively. Subsequently, non-English publications as well as manuscripts published before 1999 were excluded from further analysis. All remaining publications were screened and manuscripts relevant for the current study were selected for further analysis (Figure 1).

## 4. Conclusions

Today, surgical resection has become the standard therapy for single brain metastases in patients with a good performance status and controlled extra-cranial disease [12]. Importantly, this approach is evidence-based because randomized controlled trials have revealed prolonged survival upon surgery with WBRT compared to WBRT alone [10,70,71]. Surgical resection of distant metastases in gastrointestinal cancer (mostly liver and lung metastases) is still discussed controversially and depends (i) on the primary tumor entity; (ii) on the number of metastases and (iii) whether complete removal (R0 resection) is feasible. For colorectal cancer, resection of lung and liver metastases has been shown to prolong survival and has become standard therapy. Of note, occurrence of distant metastases does not denote a palliative situation in colorectal cancer (anymore) and metastatic surgery is performed within a curative therapeutic concept [72]. In contrast to these positive clinical developments, for esophageal, gastric and pancreatic cancer, clear evidence for the benefit of surgical resection of liver and lung metastases has not been provided (yet). Consequently, therapy for these patients mostly remains palliative and distant metastases are usually not treated surgically. Importantly, however, for all three cancer entities reports have been accumulating within the last decade which revealed that resection of distant metastases may be beneficial in certain subsets of patients [73,74,75]. In line with this consideration, most studies discussed in this review found that also surgical treatment of brain metastases originating from these cancers may prolong patient survival. In light of the fact that, at least for esophageal, gastric and pancreatic cancer, surgical resection of distant (lung and liver) metastases is generally not advised, it is remarkable that a substantial percentage of patients diagnosed with brain metastases originating from these cancers were subjected to surgical treatment. It is tempting to speculate that this fact can be (at least partially) attributed to the fact that brain metastases, in contrast to lung and liver lesions, commonly cause devastating symptoms and surgical treatment is considered to alleviate those. At the same time, this symptom-relieving therapy may not only reduce morbidity but also positively affect mortality, in some cases even providing the chance for a cure. Nevertheless, given the peculiarity of brain metastases originating from these cancers, valid data emerging from randomized controlled clinical trials for the justification and/or general recommendation of whether surgical resection of brain metastases from gastrointestinal cancers should be performed is not available to date. Moreover, it should be taken into account that surgical resection of brain metastases is more likely to be considered in patients with a single brain metastasis, patients in good conditions and absence or limited extra-cranial disease, factors which are already associated with improved prognosis. Therefore, the value of surgical resection of brain metastases from gastrointestinal cancer is not yet evidence-based and will therefore remain controversial unless larger studies will shed light on this matter. Furthermore, it should be taken into account that data about potential morbidity and mortality caused by the surgical removal of brain metastases originating from gastrointestinal cancers has not been reported to date, but, however, should potentially be considered. As a future perspective, it will be decisive to identify prognostic factors which will allow identifying patients with gastrointestinal cancers which will benefit from an aggressive therapy when brain metastases are encountered. Most likely, these prognostic factors will include clinical parameters but will also incorporate other factors such as genetic make-ups of these cancers. However, until these data are available, the decision about therapeutic approaches for brain metastases originating from gastrointestinal cancers will be made in a case-specific manner and will also incorporate the findings of the clinical reports reviewed here. Given that some reports have demonstrated prolonged (and sometimes even long-term) survival of patients receiving aggressive treatment of brain metastases from esophageal, gastric, pancreatic and colorectal cancer [45,76,77,78], considering surgical resection of brain metastases for these patients appears to be justified. In some cases, this approach may (only) tend to relieve symptoms within a palliative setting; however, in others it may also prolong survival and/or even constitute part of a curative treatment regime in a selected patient cohort.
